# Is There a Threshold for Medication Adherence? Lessons Learnt From Electronic Monitoring of Drug Adherence

**DOI:** 10.3389/fphar.2018.01540

**Published:** 2019-01-09

**Authors:** Michel Burnier

**Affiliations:** Hypertension Research Foundation, University of Lausanne, Lausanne, Switzerland

**Keywords:** hypertension, percentage days covered, cardiovascular, blood pressure, pharmacology, genotype

## Abstract

Medication adherence is a well-recognized problem in the management of patients with chronic diseases needing a long-term pharmacotherapy. While fighting against non-adherence, an important question frequently arises, i.e., how much adherence is enough to obtain the full treatment benefits? Most studies having attempted to answer this question have used large pharmacy records and health care databases to quantify the percentage of days covered by the prescribed treatment and hence deduce a threshold below which there appears to be fewer benefits from therapy. In the present paper, the use of data obtained using electronic monitoring of adherence is discussed as another means to assess adherence thresholds with a particular emphasis on hypertension. The data show that even with the use of electronic monitoring of adherence, which provides a comprehensive dosing history, it is extremely difficult to define an adherence threshold in hypertension. This is due to many factors that need to be taken into account, including not only the pattern of patients’ adherence and their clinical and environmental characteristics, but also the pharmacological characteristics of the prescribed drugs, the severity of the disease and many others. To determine adherence cut-offs more precisely, specific protocols should be designed to answer the question in various clinical conditions. These protocols should be conducted in well-defined patients’ groups, they should use the most reliable methods to measure adherence providing if possible a detailed dosing history perhaps combined with drugs levels in blood or urine. These studies should also choose the best methods to measure clinical endpoints, such as ambulatory blood pressure monitoring or home blood pressure in the case of hypertension. One important aspect is that datasets should be solid and large enough to be able to analyze adherence data as a continuous variable using newly developed mathematical models including new metrics catching the complexity of adherence. The rapid development of new technologies like devices, connectivity, and analytics, will probably provide new solutions to improve our ability to define valid and clinically useful adherence thresholds in various therapeutic areas.

## Introduction

To maintain a high level of medication adherence over years is one the most difficult challenge in the management of medical conditions, which need chronic ambulatory pharmacotherapies. This issue is even more relevant in asymptomatic conditions such as the treatment of cardiovascular risk factors. Indeed, in patients with hypertension or dyslipidaemia, non-adherence to medications is highly prevalent (Naderi et al., 2012). This is certainly one reason why many patients with a high cardiovascular risk do not benefit from otherwise effective medicines.

According to the last official consensus definition of medication adherence, adherence is the process by which patients take their medications as prescribed (Vrijens et al., 2012). This process implies three components: the initiation, the implementation, and the discontinuation. The initiation process is crucial and non-initiation may affect up to 20% of patients (Fischer et al., 2011). In hypertension, depending on its severity, non-initiation may have important clinical consequences with a high risk of serious events such as stroke or heart failure. At this stage, the main unresolved questions are: why are patients not starting their treatment and how quickly should patients initiate their therapy? However, in clinical practice, the implementation, representing the extent to which the patients’ actual dosing is in accordance with prescribed dosing regimen, and the persistence, which represents the time between initiation and the last dose, are probably the most critical parameters to estimate drugs utilization in relation with their clinical impact. Therefore, medication persistence will be the main parameter considered in our discussion.

Among all terminologies and parameters used to define adherence to medications, none enables to answer the important clinical question, i.e., how much medication adherence is enough. This is indeed a very difficult question to answer because the benefits of a good adherence may depend on several factors. These include: the pattern of non-adherence, the pharmacology of drugs, the type and characteristics of diseases (severity, duration,..), the outcome measures chosen to assess the clinical benefits (for example intermediate endpoints or hospitalizations or hard endpoints such as stroke, heart failure, or death), and of course the characteristics of patients and their social and familial environment.

In this article, the concept of “how much medication adherence is enough” is discussed in the light of two different approaches. The first is the use of data collected from electronic health records or pharmacy dispensing databases, which allow quantifying adherence as treatment discontinuation or overall persistence rate. The second is the use of data obtained from electronic monitoring of medication adherence using for example the Medication Event Monitoring System (MEMS), which provides a dosing history with a real-time capture of the dosing events.

## Health Care and Pharmacy Databases and Adherence Thresholds

In the last 10–15 years, many large regional and national health care administrative databases based on medical registries and pharmacy records have been created enabling to follow medications’ prescriptions and their discontinuation, i,e., failure to renew a prescription for a prolonged time interval, in large groups of individuals over years. With these data, it has become possible to assess indirectly the medication persistence calculating the medication possession ratio (MPR), which is the ratio of the number of drug doses taken to the number of doses prescribed over a given time period (Steiner and Prochazka, 1997), as well as the percentage of days covered (PDC) by the prescriptions. In the field of cardiovascular medicine, several analyses have demonstrated that higher MPR or PDC are associated with a lower mortality (Simpson et al., 2006) and a reduced risk of cardiovascular complications such as stroke, congestive heart failure, or coronary events (Perreault et al., 2008, 2009, 2010, 2012; Mazzaglia et al., 2009; Corrao et al., 2010, 2011, 2015; Degli Esposti et al., 2011; Qvarnstrom et al., 2013; Xu et al., 2017; Yang et al., 2017). Conversely, interrupting cardiovascular protecting drugs is associated with an increasing risk of developing such complications (Corrao et al., 2012). Similar observations have been done in other fields of medicine such as HIV therapies (Paterson et al., 2000), immunosuppression in transplantation (Dobbels et al., 2004) and recently oncology (Bhatia et al., 2015).

**FIGURE 1:**
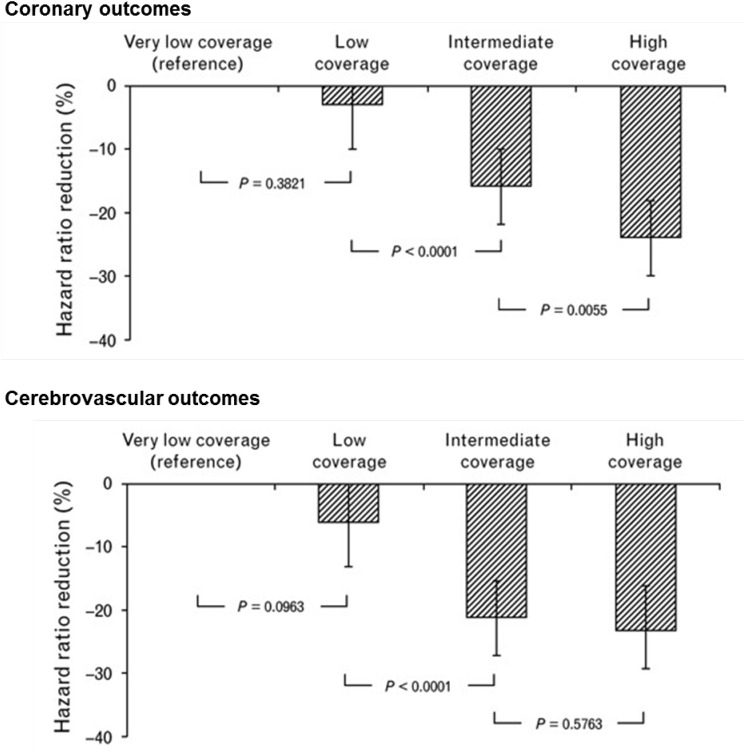
Dose-dependent effect of adherence with antihypertensive drug therapy on the reduction in hazard ratio of coronary and cerebrovascular outcomes. Analysis of the Lombardy database. Adherence measured according to the proportion of days of observation covered by antihypertensive medication. Adherence levels are the following: very low coverage: <25%; low coverage: 26–50%; intermediate coverage: 51–75%; and high coverage: >75%. Adapted from Corrao et al. (2011).

In almost all adherence analyses based on MPR or PDC, patients are distributed in arbitrarily defined categories of adherence such as for example: ≥80% and <80%, or ≥80%, ≤50 to <80% and <50% without a specific scientific rational or meaning for these choices. With such categorizations, authors usually find a dose-effect relationship between the percentage of adherence and chosen endpoints, a higher adherence being associated with better outcomes as discussed above. Figure 1 illustrates one example of the relationship between adherence to antihypertensive therapy and the risk of coronary and cerebrovascular events in 445,356 patients from the Lombardy database (Corrao et al., 2011). However, it is interesting to note that almost all these studies used the arbitrary threshold of 80% to characterize a good adherence as proposed by R.B Haynes in his book on adherence published together with DL. Sackett in 1976 (Haynes, 1976). Yet, in addition to being arbitrarily defined, this threshold is not always supported by research documenting the appropriateness of cutoffs for specific medication classes or diseases (Cramer et al., 2008). Thus, in five cohorts of patients diagnosed with schizophrenia, diabetes, hypertension, congestive heart failure, or hyperlipidemia, the optimal cut-off adherence values for the MPR and PDC in predicting disease-specific hospitalizations across the five cohorts ranged from 0.58 to 0.85 (Karve et al., 2009). In lipid management, direct comparisons of the 0.9–1.0 MPR category versus the 0.8–0.89 MPR category demonstrated a significant increase in odds of achieving 25% or more reduction in total cholesterol, LDL and non-HDL in the highest category (Watanabe et al., 2013). In fact, estimations of the relationship between adherence and outcomes based on the MPR or the PDC have several limitations. Firstly, most studies have not been designed with the goal of defining prospectively an adherence threshold. Hence, values remain rough estimates. Secondly, the methods used to measure adherence are very indirect and authors have no idea on what patients actually did with their medications during the period of observation. Thirdly, authors do not take into account that a mean of 80% can represent several different patterns of adherence such as missing one day on five or one month on five, the clinical impact being very different in each condition. Fourthly, the quality of outcome measurements is not always optimal. For example, in hypertension, the main outcome is often office blood pressure measured only once by physicians. Home blood pressure or 24 h ambulatory blood pressure monitoring would be more reliable and hence appropriate. At last, several different methodologies were used to estimate the cut-off points including multiple logistic regressions, univariate logistic regression models, and calculation of the upper most left point of the receiver operating curve corresponding to the maximum specificity and sensitivity of the adherence value (Karve et al., 2009; Oleen-Burkey et al., 2011; Watanabe et al., 2013). These statistical methods present some limits as they do not include the major factors affecting adherence and they do not take into account the fact that the relationships between adherence and clinical endpoints may not always be linear. Thus, MPR and PDC are concepts, which provide statistically calculated threshold values but their clinical validity should be confirmed using other approaches. For example, one often neglected aspect is the variable nature of adherence over time as mentioned above. Finally, these analyses do not assess the specific thresholds of drug or drug classes but rather the clinical impact of poor adherence to a global treatment. Finally, when considering only one threshold (for example 80%), these analyses do not envisage that they may differ substantially from one disease to another.

Taken together, these observations on the ability to define adherence thresholds using large administrative or health care databases suggest that these records have the advantage of collecting data from very large unselected groups of patients over a long period of time. They can produce interesting information on drug prescriptions and withdrawals, and therefore on the long-term persistence with the various drug classes. These datasets represent also unique opportunities to analyze the relationship between the adherence pattern and hard clinical outcome parameters such as stroke, cardiovascular events of death since they are connected to the patients’ clinical history. Yet, they provide only a rough estimate of how much medication adherence is necessary to obtain the expected clinical benefits of a therapy. This limitation is due essentially to the fact that neither MPR nor PDC make use of the patients’ dosing history. In the future, newer technologies enabling to acquire dosing histories coupled electronically to clinical data (see below) might overcome some of the limitations of MPR and PDC. The development of more complex statistical models, with the addition of new metrics reflecting the multiple facets of the adherence phenomenon, may also help improving the assessment of valid thresholds using MPR or PDC.

## Defining an Adherence Threshold Using Data Collected by Electronic Monitoring of Adherence

The development of the MEMS in the 1970s was a major breakthrough in the field of adherence. Indeed, for the first time, it became possible to follow a patient’s medication adherence on a day-to-day basis, and hence to obtain dosing histories for long periods. To date, more than 750 scientific papers have been published using this methodology, involving more than one million trial subjects. Conceptually, electronic monitoring of drug adherence is also an indirect measure of adherence, as one cannot assure that once the bottle is opened, the dose is taken. However, studies have confronted the MEMS data with plasma drug concentrations and 97% accuracy was found between drug concentrations and openings of the monitor and time of ingestion of the prescribed dose. This suggests that the instances where the pillbox is opened and the drug not taken are relatively exceptional (Vrijens and Urquhart, 2014). Therefore, today, one can consider the use of the MEMS as the gold standard to measure adherence. However, other systems become available, such as the Proteus Digital Health system, which enable to catch simultaneously the complete dosing history and the proof that the drug was ingested (Belknap et al., 2013; DiCarlo et al., 2016). The system consists on an ingestible nanosensors incorporated in the pill during the manufacturing process. Once ingested, the sensor is activated in the stomach and generates a message coded for the medication name and dose that is transmitted to a wearable patch and then to a designated device. This new system may be very helpful to describe more adequately the long-term relationship between adherence and clinical outcomes. However, as of today, there are not enough data available to discuss it in more details in this paper.

Nevertheless, with the use of the MEMS in clinical studies and clinical practice, we have learnt enormously on the multiple facets of medication adherence that may affect the determination of adherence thresholds. In the field of hypertension, these include: the high variability of adherence patterns within and between individuals, the inconsistency of the relationship between drug adherence and clinical endpoints such as blood pressure control, the variable pharmacological profile of antihypertensive drugs and particularly the inconsistent relationships between plasma drug levels and clinical effects. These different issues will be discussed below.

### Adherence Is a Dynamic Process

The first lesson learnt from analyses of the dosing histories is that drug intake is extremely variable in all clinical conditions and that adherence is a dynamic process, which one cannot summarize with a single figure. Indeed, adherence can be perfect during a certain period and erratic during another one. Deviations from the prescribed regimen are very frequent and are characterized by isolated or sequential omissions, drug holidays or compensatory overdosing (Burnier et al., 2013). This means that to define a threshold, the pattern of drug omissions should be taken into account. Averaging different periods of good and poor adherence might be of interest, but the meaning of such averages to establish a threshold would need more validations. The element of variability in non-adherence patterns over time increases the complexity of defining a valid cut-off point using traditional regression analyses, which never include such component in the list of relevant variables.

### Relationship Between Adherence and Outcome

**FIGURE 2:**
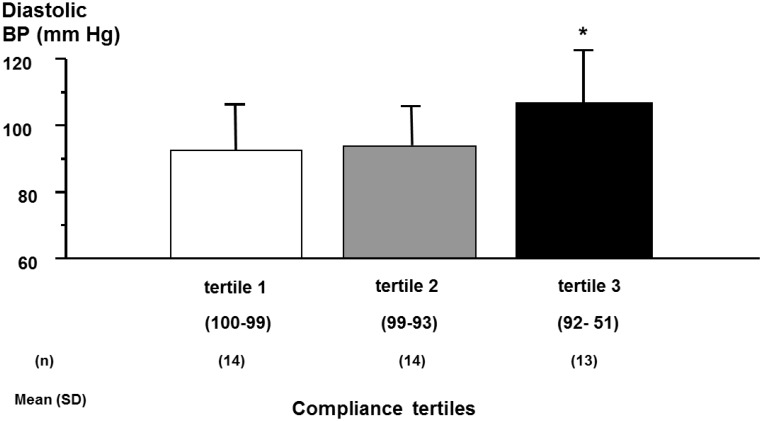
Achieved diastolic blood pressure (BP) according to compliance tertiles in patients with resistant hypertension. Achieved 12 h daytime ambulatory diastolic blood pressure according to tertiles of compliance during the first 2 months of compliance monitoring (ANOVA: *F* = 3.52, *P* = 0.039). ^∗^*P* < 0.05 versus tertile 1. Adapted from Bertholet et al. (2000).

In hypertension, one can correlate the level adherence with the reduction in blood pressure (BP) or the occurrence of cardiovascular complications or death. There have been several attempts to characterize the relationship between drug adherence and the reduction of BP or the number of subjects reaching the recommended target BP, with the hope to demonstrate that the better the adherence to antihypertensive therapy, the lower the BP. However, in general, these analyses were not done with the main goal to define adherence thresholds. In 2004, Wetzels et al. (2004) made an excellent review of about 30 studies, which analyzed this aspect in hypertension using the MEMS system to quantify drug adherence. Results were quite disappointing with a high heterogeneity, some authors finding a significant correlation between BP reductions or BP control and the level of adherence (Vaur et al., 1999; Wetzels et al., 2004) and some not (Mallion et al., 1996). Moreover, even when a correlation was found, this latter was rather weak. Interestingly, a significant dose-response was observed when data were analyzed according to predefined categories of adherence, whereas no correlation was found when data were categorized according to categories of achieved BP (Wetzels et al., 2004). Results were different depending on the clinical characteristics of the hypertensive population. Thus, in a group of patients with resistant hypertension receiving at least a triple therapy, the percentage of patients with an achieved diastolic BP < 90 mmHg was lowest in patients having an adherence rate between 51 and 92%, when patients were analyzed by tertiles to define the cut-off below which BP was uncontrolled (Burnier et al., 2001; Figure 2). These findings suggest that, in resistant hypertension, the threshold is not at 80% as initially anticipated but rather at >92%. In contrast, in another set of 60 treated hypertensive patients who did not respond to a bi-therapy, one could not observed a difference in adherence level between patients who reached the BP targets of <140/90 mmHg and those who improved but did not normalized their BP and those who did not modify their BP (Bertholet et al., 2000).

These contrasting results may be explained in several ways. Firstly, adherence to therapy improves as soon as it is measured (Hawthorne effect). Patients participating in studies receive a greater attention, which increases their adherence, thereby blunting the difference between the studied groups. Secondly, in hypertension, none of the drugs enables to control BP in 100% of subjects, even if the adherence is perfect. Thirdly, the dose-response curve of most antihypertensive drugs, except loop diuretics, is rather flat. Therefore, the impact of non-adherence on BP is often confounded by poor responses to the prescribed drugs and the absence of dose-response. In fact, when assessing the relationship between adherence and BP control, four patterns can occur: patients may be fully adherent with a good BP response or fully adherent with a poor BP response, if drug therapy is inadequate. However, some patients may be poorly adherent with still a good BP response, for example if they are over-treated, or non-adherent with no BP response. This indicates once again that individual patterns play an important role and may limit the ability to define an adherence cut-off point. Fourthly, in most studies reviewed by Wetzels et al. (2004), subjects were receiving several antihypertensive drugs. Therefore, the analyses examined the threshold of adherence to a treatment strategy rather than to a given drug.

### Impact of the Pharmacological Profile on the Correlation Between Adherence and Outcome

The pharmacological profile of drugs (absorption, distribution, metabolism, and excretion) has a major impact on the relationship between the level of adherence and clinical outcomes. Indeed, the impact of missed doses or transient discontinuations on clinical outcomes is influenced by the dose-response curve of the prescribed drug, its duration of action, and its metabolism. For example, in an analysis comparing three antihypertensive drugs with different half-lives, we have demonstrated that drugs with a longer duration of action have a better profile to reduce the risk of complications in patients with hypertension when adherence is suboptimal (Burnier et al., 2011). It has also been possible to demonstrate, using specifically designed protocols, that drugs with a longer duration of action such as amlodipine or aliskiren attenuate the impact of missed doses on BP (Leenen et al., 1996; Palatini et al., 2010). Consequently, multiple isolated drug omissions with these agents might have little if any influence on clinical endpoints. These latter studies demonstrate not only the important role of drugs’ duration of action on clinical outcomes when patients are poorly adherent, but also that it is possible to design specific clinical protocols to answer a simple, but clinically relevant, question such as “what is the impact of missed doses ?”

Other drug properties and/or patients’ characteristics may play a critical role in the possibility to define an adherence threshold. One of them is the pharmacogenetic profile influencing the metabolism and elimination of drugs. Thus, in a recently published study, we compared the inhibition of platelet aggregation induced by three different anti-P2Y12 drugs, clopidogrel, prasugrel, and ticagrelor, in relation to drug adherence measured with the MEMS in 120 patients with coronary heart diseases and a coronary stent (Forni Ogna et al., 2017). In this study, mean adherence was very high with all three compounds, respectively, 100, 100, and 96% for clopidogrel, prasugrel, and ticagrelor. However, at 6 months, the degree of inhibition of platelet aggregation, measured using the VASP-PRI, was very different, respectively, 17.7 ± 11.0% with ticagrelor, 29.2 ± 15.5% with prasugrel and 47.2 ± 17.6% with clopidogrel. With clopidogrel, platelet inhibition was unpredictable and extremely variable, independently of the patients’ adherence. Indeed, the main determinant of the clinical response to clopidogrel was the CYP2C19 genotype (Forni Ogna et al., 2016). Consequently, in patients receiving clopidogrel, adherence correlated with the VASP inhibition only in those patients carrying defined CYP2C19 genotypes. This observation further illustrates the complexity of defining a global threshold that may be valid for all individuals.

Of course, the best situation to characterize an adherence threshold is when there is a close correlation between plasma drug levels and clinical endpoints. This is the case, for example, of immunosuppressive agents in transplantation or drugs in HIV therapy, clinical situations in which therapeutic drug monitoring is performed, but definitively not in hypertension. Thus, in HIV therapy, Paterson et al. (2000) assessed the effects of different levels of adherence to therapy on virologic, immunologic, and clinical outcomes in 99 patients treated with a protease inhibitor. Adherence, measured with the MEMS, was significantly associated with successful virologic outcome and increase in CD4 lymphocyte count. However, the most important observation made in this study is that virologic failure occurred significantly less in patients with adherence of 95% or greater (22% failures) than in those with 80–94.9% adherence (61% failures), and those with less than 80% adherence (80% failures). These data suggested that the adherence threshold necessary to obtain the full treatment benefits is very high in this patients’ population. A similar observation was done in patients with resistant hypertension (Burnier et al., 2001).

Another example is the use of adalimumab in the treatment of patients with psoriasis, rheumatoid arthritis or inflammatory bowel diseases. In this case, serum levels of adalimumab have been reported to correlate with clinical outcomes such as remission of inflammatory bowel disease or a reduction in articular symptoms in patients with rheumatoid arthritis, with 4.9 mg/L as the therapeutic threshold of adalimumab serum concentration (Bartelds et al., 2007; Roblin et al., 2014). In a small study involving 7 patients with moderate to severe psoriasis, a pharmacokinetic model has been applied to serum drug levels in relation to adherence data obtained with the MEMS (Morrison et al., 2015). With this approach, the authors have demonstrated that it is possible to define objectively a threshold for adherence based on a therapeutic drug serum level and MEMS data, thus avoiding the use of an arbitrary cutoff. A recent analysis of 6048 patients treated with adalimumab for inflammatory bowel diseases supports this hypothesis (Govani et al., 2018). In this patient group, the optimal threshold was 86%, as defined with the MPR.

In hypertension, this approach is much more difficult to apply because the relationship between plasma drug levels and the therapeutic outcome – i.e., the reduction of BP – is usually weak. Thus, in 1980 already, Waeber et al. (1980) demonstrated that there is a large discrepancy between the sustained antihypertensive effect of captopril and its short duration of inhibition of the angiotensin converting enzyme, as an indirect marker of circulating drug levels. With angiotensin receptor blockers, we have also demonstrated that protein binding and the affinity for the angiotensin AT1 receptor are important determinants of the BP lowering effect of these drugs (Maillard et al., 2001). Thus, the amount of drug bound to the AT1 receptor is more relevant than plasma drug levels to explain the antihypertensive efficacy of these compounds. Therefore, with this type of drugs, the determination of a therapeutic threshold *based* on plasma drug levels is difficult as will be the definition of an adherence cut-off value.

## Conclusion

Taken together, these studies demonstrate that dosing histories, obtained with the use of an electronic monitoring system, might improve our ability to define adherence thresholds for various drug therapies. In some circumstances, electronic monitoring of adherence may be superior to calculation of the PDC or MPR, but in other situations, it is still insufficient. One example is the assessment of the relationship between adherence and hard clinical endpoints such as cardiovascular events and death in hypertension or dyslipidemia. Indeed, many studies performed with the MEMS in these indications were of too short duration to conclude on hard end-points. Yet, when the MEMS is used in phase 2/3 clinical trials, it is a very potent tool to link adherence to outcomes and this has been demonstrated essential in the development of HIV drugs (Tolley et al., 2010). Today, none of the available methods to measure adherence seems to be fully adequate to determine adherence cut-offs but this may change in the future with the availability of the Proteus Digital Health system, which provides the dosing history and the proof of ingestion, provided long-terms studies are conducted with this system. Besides the reliability of the adherence measurements, one major challenge remains the large variability of pharmacological parameters within patients’ populations. In fact, very few clinical and pharmacological studies have examined prospectively the adherence threshold below which a drug may clearly become ineffective. Today, when the dose-response relationship of a new compound is investigated, drug adherence is rarely taken into account because investigators assume that adherence is perfect in phase 2 and 3 studies, an assumption, which may not necessarily be correct (Burnier and Wuerzner, 2015). In our opinion, adequate adherence measures should be included in all steps of drug development. Today this can be done easily either with the MEMS or with the Proteus Digital Health system. The FDA has accepted these systems for such purposes. These methods may even be combined with the determination of plasma or urinary drug levels, which have been shown to be useful for example in the management of resistant hypertension (Hamdidouche et al., 2017).

One should also consider designing specific prospective protocols to evaluate adherence thresholds, as it was done to assess the impact of missed doses. In these protocols, the study population should be as homogenous as possible to provide reliable and applicable answers. Adherence measures should be considered as critical parameters and statistical plans should include analyses according to the level of adherence as a continuous variable. Statistical models should be ameliorated to capture the impact of the multiple factors affecting adherence, as discussed previously. Adherence-adjusted analyses offer the potential for more powerful tests and better understanding of trial data and their clinical implications including the determination of adherence thresholds. Pre-randomization factors associated with good trial-related product adherence should be identified and adherence-adjusted analyses be considered early in the planning of clinical trials. The rapid development of new technologies like devices, connectivity and analytics, and the further development of healthcare databases will probably help finding new solutions to improve our capacity to answer complex questions such as “how much adherence is enough.” Thus, there will be a lot of interesting new clinical research to conduct, which will enable to fly with better instruments!

## Author Contributions

The author confirms being the sole contributor of this work and has approved it for publication.

## Conflict of Interest Statement

The author declares that the research was conducted in the absence of any commercial or financial relationships that could be construed as a potential conflict of interest.
